# The PKS–NRPS Gene *BBA_09856* Deletion Mutant of *Beauveria bassiana* Enhanced Its Virulence Against *Ostrinia furnacalis* Larvae and Strengthened the Host Plant’s Resistance to *Botrytis cinerea* as an Endotype

**DOI:** 10.3390/jof11030197

**Published:** 2025-03-04

**Authors:** Yanan Wang, Xiaowei Zou, Xiaomin Zhu, Ji Qi, Jianfeng Liu, Zhengkun Zhang

**Affiliations:** 1 College of Life Sciences, Jilin Normal University, Siping 136000, China; yanan1121v@163.com (Y.W.);; 2 Key Laboratory of Integrated Pest Management on Crops in Northeast China, Ministry of Agriculture and Rural Affairs, Institute of Plant Protection, Jilin Academy of Agricultural Sciences, Changchun 130033, China; zouxiaowei2008@126.com (X.Z.); zxm5210@126.com (X.Z.)

**Keywords:** *Beauveria bassiana*, entomopathogenic fungi, nonribosomal peptide synthetase, polyketide synthase, mycoinsecticides

## Abstract

Nonribosomal peptide synthetase (NRPS) and polyketide synthase (PKS) play crucial roles in the development and pathogenicity of the entomopathogenic fungus *Beauveria bassiana*. However, they are among the few biosynthetic gene clusters with unknown functions in *B. bassiana*. To investigate the role of the hybrid PKS–NRPS synthetase gene *BBA_09856* in *B. bassiana*, we constructed a mutant strain, ∆*BBA09856*-WT, by deleting the *BBA_09856* gene through Agrobacterium-mediated transformation. We then analyzed the biological characteristics of the mutant strain and the virulence of the mutant strain toward *Ostrinia furnacalis* larvae, as well as its antagonistic effects against the phytopathogen *Botrytis cinerea*. We found that the average growth rate of the three mutant strains, ∆*BBA09856*-WT, was significantly higher compared to the wild-type (WT) strain on the 15th day of culture on potato dextrose agar (PDA) plates (7.01 cm vs. 6.30 cm, *p* < 0.01). Additionally, the average spore production(3.16 × 10^7^/cm^2^ vs. 9.95 × 10^6^/cm^2^, *p* < 0.001) and germination rate (82.50% vs. 54.72%, 12 h, *p* < 0.001) were significantly different between the three mutant strains, ∆*BBA09856*-WT, and the WT strain. The average survival rates of *O. furnacalis* infected with the WT strain and the three mutant strains, ∆*BBA09856*-WT, after 8 days were 61.66%, and 30.00%, respectively, indicating that the pathogenicity of the tested mutant strains was significantly greater than that of the WT strain. The results of the dual culture test indicated that the inhibitory rates of the WT and ∆*BBA09856*-WT strains against *B. cinerea* were 40.25% and 47.65%, respectively (*p* < 0.001). Similarly, in the dual culture test, the WT strain reduced the growth of *B. cinerea* by 9.90%, while the ∆*BBA09856*-WT exhibited a significantly greater inhibition rate of 28.29% (*p* < 0.05). The diameters of disease spots, measured 6 d after inoculation with *B. cinerea* in the tomato treatment groups, revealed significant differences in endophytic colonization between the WT and ∆*BBA09856*-WT strains in the WT+Bc and ∆*BBA09856*-WT+Bc treatment groups (15.26 mm vs. 12.16 mm, *p* < 0.01). Notably, ∆*BBA09856*-WT exhibited enhanced virulence toward *O. furnacalis* larvae and increased antagonistic activity against *B. cinerea*. Our results indicate that the gene *BBA_09856* may have a negative correlation with the development and virulence of *B. bassiana* toward the insect pest *O. furnacalis* larvae, as well as its antagonism against *B. cinerea*. These findings suggest that molecular techniques, such as gene editing, could be employed to develop superior strains of *B. bassiana* for the biological control of plant diseases and insect pests.

## 1. Introduction

Entomopathogenic fungi (EPF) are specialized microorganisms that can infect arthropods. To date, over 750 species of EPF, classified into 85 genera, have been documented that have the ability to infect more than 1000 insect pest species [1,2]. The genera *Beauveria* spp. and *Metarhizium* spp. are readily cultivated fungi with extensive host ranges that have been utilized as biocontrol agents against diverse pests [3,4,5,6]. *B. bassiana*, a globally distributed fungal pathogen of insects, has been extensively studied in relation to its pathogenesis, emerging as a prominent model to explore pathogen–host interactions [7]. However, there are barriers to the application of *B. bassiana* as a biocontrol agent, including prolonged pest control cycles and the loss of virulence [8,9]. Investigating the roles of virulence-associated genes in *B. bassiana* to develop enhanced fungal strains through genetic engineering represents a critical strategy to overcome these challenges [10,11].

There are three main stages in *B. bassiana* infection of insects. The initial phase begins with the attachment of conidia onto the epicuticle, where they germinate and develop into an invasive appressorium. This fungal structure generates turgor pressure by converting lipid droplets into glycerol, which supplies the necessary force to penetrate the host cell wall [1,12]. At the same time, it releases enzymes such as proteases, chitinases, and lipases that degrade the host’s cell wall, enabling fungal invasion [13,14]. Once the integument has been penetrated, *B. bassiana* colonizes the hemolymph and produces a variety of bioactive secondary metabolites that are essential for its pathogenesis and virulence. Subsequently, as the insect succumbs and hemolymph nutrients are depleted, the circulating blastospores differentiate into hyphal morphologies. These then emerge on the exterior of the insect for subsequent sporulation on the host surface [1,15,16]. *B. bassiana* predominantly produces insecticidal toxins, including beauvericin and bassianolide, which are classified as nonribosomal peptide compounds, as well as oosporein, a polyketide compound [17,18]. Recent advancements in whole genome sequencing technologies and bioinformatics have uncovered numerous biosynthetic gene clusters (BGCs) that might play a role in the production of bioactive compounds, including insecticides, immunosuppressors, and antimicrobials. In BGCs, the genes encoding biosynthetic enzymes are involved in the production of secondary metabolites. These enzymes include nonribosomal peptide synthetases (NRPSs), polyketide synthases (PKSs), and PKS–NRPS hybrid synthetases [1]. The genome of *B. bassiana* ARSEF 2860 harbors 21 NRPS or NRPS-like genes, which play crucial roles in the biological characteristics and pathogenicity of the fungus. Liu et al. revealed that the genes *BBA_03671*, *BBA_04028*, and *BBA_08222*, which encode NRPSs, are significantly involved in the process of insect infection [18]. By contrast, although *BBA_06727* does not directly contribute to insect infection, it influences the growth and development of the fungus following infection [18]. Polyketide synthases are essential enzymes containing multiple functional domains utilized in the biosynthesis of polyketides. Polyketides represent a broad class of secondary metabolites known for their diverse biological activities and pharmacological properties, serving as regulators of the host–fungi interaction. They influence various processes such as sporulation, melanization, stress responses, and signal transduction [3]. Previous research has demonstrated that four *pks* genes, namely *pks9* (*OpS1*), *pks14*, *pks15*, and *BbpksP*, are important for virulence against insects in *B. bassiana* [7,19,20,21,22]. Furthermore, the deletion of *BbpksP* resulted in reduced colony size and conidial germination following exposure to UV-B radiation. Additionally, this deletion altered the ultrastructure of the cell wall [7]. The deletion of *pks15* resulted in a modest inhibition of radial growth, accompanied by a significant reduction in sporulation and the presence of abnormal cell wall organization [21,22]. In contrast, deletion of *pks9* did not produce any significant effects on fungal growth or tolerance to oxidative stress [19]. These findings confirmed the significance and diverse roles of *pks* genes in fungal development, environmental adaptation, and the biocontrol potential of *B. bassiana*. However, the functions of additional gene clusters encoding PKS, NRPS, and PKS–NRPS have yet to be fully determined.

*Beauveria bassiana* not only directly infects pests, but also endophytically colonizes plants through various inoculation methods. This interaction establishes a symbiotic relationship with the plants, which, in turn, enhances their growth and strengthens their resistance to insect pests and pathogens [23,24,25]. For instance, the reduction in tunneling activity caused by *Ostrinia nubilalis* and *Sesamia calamistis* in maize was attributed to the successful endophytic colonization of *B*. *bassiana* in corn [26,27]. And numerous studies have established a link between the decrease in damage inflicted by insect pests and the accumulation of mycotoxins within plant tissues. It is clear that the endophytic colonization of plants by *B. bassiana* significantly enhances the plants’ resistance to diseases and insect pests. However, the regulatory pathways that govern the synthesis of insecticidal toxins and metabolic products in *B. bassiana*, along with the associated genes, have not yet been fully identified.

This study primarily investigated the role of the PKS–NRPS gene *BBA_09856* in the development of the *B. bassiana* and its virulence against *Ostrinia furnacalis* larvae, as well as its effects on tomato resistance to *B. cinerea* following colonization by *B. bassiana*. The aim was to elucidate the function of the PKS–NRPS gene *BBA_09856*. Our findings are expected to provide fundamental insights into the mechanisms underlying the interaction between *B. bassiana* and the plant pest *O. furnacalis*, as well as the gray mold disease caused by *B. cinerea*. Additionally, this research may contribute to the development of superior fungal strains for plant pests and diseases control.

## 2. Materials and Methods

### 2.1. Sources of Insect and Fungal Isolates

*B. bassiana* OFDH1-5 (preservation number: ACCC32726) and the insect pest *O. furnacalis* larvae, were obtained from the Institute of Plant Protection at the Jilin Academy of Agricultural Sciences, Changchun City, Jilin Province, China. The *B. cinerea* was provided by Prof. Wei Li, College of Plant Protection, Hunan Agricultural University, Hunan, China. *Escherichia coli* DH5α (Sangon Ltd., Shanghai, China) was cultured in Luria Bertani medium (Sangon) at 37 °C and utilized for vector construction. *Agrobacterium tumefaciens* AGL-1 (Sangon Ltd., Shanghai, China), used for fungal transformation, was cultured at 28 °C in *Agrobacterium* rhizogenes liquid medium (YEB).

### 2.2. Extraction of Genomic DNA from B. bassiana

The wild-type *B. bassiana* strain was inoculated onto potato dextrose agar (PDA) plates and incubated at 25 °C for 7 d. Subsequently, the mycelia were harvested from the plates, and DNA extraction was performed using the method described by Pinnamaneni et al. [28].

### 2.3. Targeted Gene Deletion

The plasmid pDHt-bar was provided by Chengshu Wang from the Shanghai Institute of Plant Physiology and Ecology, Chinese Academy of Sciences [29]. This plasmid was used as a backbone to delete the target gene. Briefly, two DNA fragments of 985 bp and 1034 bp, corresponding to the 5′ and 3′ flanking regions of the gene *BBA_09856*, were amplified using conventional PCR using the primer pairs listed in Table S1. The upstream fragment was digested using the restriction enzymes *EcoRI* and *PstI*, while the downstream fragment was digested with the restriction enzymes *SpeI* and *SacI*. These fragments were then cloned into the appropriate sites of the plasmid pDHt-bar, which contained a glyphosate resistance gene. The resulting plasmid, pDHt-*BBA09856*-up-bar-*BBA09856*-dn, was utilized for target gene disruption. The heat shock transformation method was employed to introduce pDHt-*BBA09856*-up-bar-*BBA09856*-dn into *Agrobacterium* AGL-1 [30]. The *Agrobacterium* AGL-1 strains containing the positive plasmid were selected from kanamycin resistance plates and underwent PCR amplification of a 2953 bp product using the primer pair *BBA09856*-up-F and *BBA09856*-dn-R. The PCR product was then sent to Kumei Technology Co., Ltd. (Changchun, China) for sequencing to confirm the accurate *Agrobacterium* transformants. The method for *Agrobacterium*-mediated transformation of the recombinant vector into *B. bassiana* for targeted gene deletion was performed according to the description of Fang et al. [30]. The positive transformants were transferred to the SDY medium containing 0.1% glyphosate solution and 300 μg/mL cephalothin. After being cultured at 26 °C for 7 d, the samples were subcultured three times and subsequently subjected to PCR verification using the pair of primers KObar-F and KObar-R to assess the genetic stability of the mutants. For Southern blot analysis, the bar gene (550 bp) was amplified from plasmid pDHt-bar DNA and labeled using the DIG High Prime DNA Labeling and Detection Starter Kit I (Roche, Basel, Switzerland) according to the manufacturer’s instructions. Genomic DNAs from both the wild-type strain and the mutant strains, Δ*BBA09856*-WT, were prepared as described in Section 2.2. The genomic DNAs were individually digested with *BamH*I and separated by electrophoresis in a 1% agarose gel. The DNA was transferred and cross-linked to a Hybond N+ nylon membrane (GE Healthcare Life Sciences, Marlborough, MA, USA). Membrane hybridization with the labeled bar probe was performed overnight at 42 °C. After hybridization, the membrane was stained using the aforementioned kit and developed in the dark room to detect the bands [20].

### 2.4. Plant Material Origin and Preparation

Tomato (*Solanum lycopersicum* var. Beauty) seeds were obtained from Jilin Mainland Seed Industry Co., Ltd., Gongzhuling, Jilin, China. Seeds were washed in a 1% sodium hypochlorite solution for 3 min, followed by immersion in 75% ethanol for 2 min. Afterward, they were rinsed three times with sterile water. Following surface sterilization, the seeds were sown in 10 cm × 8 cm seedling pots filled with sterilized field soil. Before planting, the soil was autoclaved twice for 2 h, with a one-day interval between autoclaving sessions. The soil was then aerated and mixed to prevent the entrapment of gases that could be toxic to microbiota and plants. The seeds were sown directly in the greenhouse located in Changchun City, Jilin Province, at a temperature of 24–26 °C, with 12 h of light and 12 h of darkness. Seedlings were watered with sterilized water at 5 to 6 intervals in the experiment [31].

### 2.5. Preparation of B. bassiana Culture and Conidial Suspension

The tested strains of *B. bassiana* were cultured on PDA at 25 °C and 80% humidity. Conidia used for infection were harvested from 3- to 4-week-old cultures by scraping the surface of the mycelium using sterile cell scrapers into sterile deionized water containing 0.1% Tween-80 [31]. The number of cells was counted using a hemocytometer to determine the conidia yield. The formula for the calculation was as follows: conidia yield (number/mL) = total cell count × dilution factor × 5 × 10^4^ [32]. The conidia were then counted and diluted to a concentration of 1 × 10^7^ conidia/mL for future use. If the experiment requires a concentration lower than this, appropriate dilutions should be made accordingly.

### 2.6. Plant Inoculation with B. bassiana and B. cinerea

The tomatoes were inoculated with the wild-type strain and the mutant ∆*BBA09856*-WT, respectively, following the method detailed by Sui et al. [31]. Subsequently, the control group tomatoes were not endophytically colonized, whereas the experimental group tomatoes were endophytically colonized by either the wild-type *B. bassiana* strain or the mutant strain ∆*BBA09856*-WT. These plants were then inoculated with *B. cinerea* under moisture-maintaining conditions, as described by Sui et al. [31].

### 2.7. The Biological Characteristics of the Mutant Strain ∆BBA09856-WT

Three genetically stable mutant strains of ∆*BBA09856*-WT were selected for the analysis of their biological characteristics.

For the growth rate test, a 2 µL suspension of *B. bassiana* at a concentration of 5 × 10^4^ conidia/mL was added to the center of each 20 mL PDA plate with a diameter of 9.0 cm. The plates were then incubated at a constant temperature of 26 °C, with 70% relative humidity (RH) and a photoperiod of 16 h of light and 8 h of darkness. The colony diameter was measured daily using the cross-method over a period of 15 d, with five replicates for each of the tested strains [32]. The colony growth rate was calculated using the following formula: colony growth rate(mm/d) = ((final colony diameter − initial colony diameter))/(culture time). Fixed plate cultures from different treatments were photographed on days 5, 10, and 15 to observe changes in fungal morphology and to compare for significant differences within each treatment group [32].

For the spore production test, a 100 µL suspension of *B. bassiana* at a concentration of 1 × 10^7^ conidia/mL was evenly spread on PDA plates. The suspension was then mixed thoroughly and incubated at a constant temperature of 26 °C for 15 d under a photoperiod of 16 h of light and 8 h of darkness. Five agar blocks, each with a diameter of 5 mm, were randomly sampled from each plate. A spore suspension was prepared by diluting the agar blocks in 5 mL of 0.1% (*v*/*v*) Tween-80 solution. Each strain was tested in triplicate, and the number of cells was counted using the hemocytometer to determine the conidia yield per unit area [32].

To evaluate the conidial germination rates of the *B. bassiana* strains, 1 mL of a suspension containing 1 × 10^7^ conidia/mL was inoculated into 20 mL of SDY liquid medium. The mixture was incubated at 26 °C with shaking for 12 h. After incubation, the spore germination was assessed using a hemocytometer, and the germination rate was calculated, ensuring that three replicates were performed for each strain [32].

### 2.8. Assessing the Virulence of the Tested B. bassiana Strains Against O. furnacalis

The three stable mutant strains of ∆*BBA09856*-WT were chosen to analyze their virulence against *O. furnacalis*.

Fungal virulence toward *O. furnacalis* was assessed using the dip method. Third-instar *O. furnacalis* larvae were immersed in 2 mL suspensions (1 × 10^7^ spores/mL) containing 0.05% Tween-80 for 20 s and subsequently reared normally on artificial feed. The larvae were divided into three groups: wild-type (WT), ∆*BBA09856*-WT, and a sterile 0.05% Tween-80 solution as a blank control. Each group consisted of three replicates, with 20 larvae in each replicate. The number of dead insects was recorded every 24 h from the second to the eighth day of inoculation with *B. bassiana*. And the mutant strain exhibiting the highest virulence toward *O. furnacalis* was selected for the following experiment.

### 2.9. Testing the Antagonistic Activity of ∆BBA09856-WT Against B. cinerea In Vitro

To elucidate the direct inhibitory effects of both the wild-type strain and the mutant strain Δ*BBA09856*-WT of *B. bassiana* on the phytopathogen *B. cinerea*, two 5 mm diameter fungal blocks from the tested strains of *B. bassiana*, cultured for 7 d, were inoculated 1.5 cm apart on either side of the fungal pathogen block along the same line. In contrast, the control group was inoculated solely with the fungal pathogen block. The plates were incubated at 26 °C and observed and photographed at 5 and 8 d post-inoculation (dpi). Each treatment was replicated five times. The colony diameter of the pathogenic fungi was measured and recorded at 5 and 8 dpi, and the inhibitory rate was calculated using the following formula [33]:

$$Inhibition\;rate\left(\%\right)=\left[\frac{\left(Control\;group\;colony\;diameter-Treatment\;group\;colony\;diameter\right)}{Control\;colony\;diameter}\right]\times 100\%$$

To evaluate the inhibitory effect of fungal metabolites on the growth of pathogenic fungi, 5 mm diameter blocks of *B. bassiana* were inoculated into potato dextrose (PD) medium. The inoculated blocks were then incubated in a constant-temperature shaking incubator at 26 °C and 180 rpm for 7 d. After incubation, the mycelia were removed using sterilized gauze and centrifuged at 16,000× *g* for 10 min at 4 °C. The supernatant was obtained by filtering through a 0.22 μm filter. This supernatant was mixed with PDA culture medium in a volume ratio of 1:5 to create the antagonistic culture medium, into which a 5 mm block of the pathogen was placed at the center. Sterilized water was mixed with PDA in a volume ratio of 1:5 to serve as the control. All plates were incubated at a constant temperature of 26 °C. The colony diameter of the pathogenic fungi was measured and recorded at 3 dpi, and the inhibition rate was calculated using the formula of the direct inhibitory effects of the tested strains. Each treatment was replicated five times [33]. The morphology of hyphae was observed and recorded using a light microscope LW300.48LT (Siwei Optoelectronic Technology Co., Ltd., Xi’an, China).

### 2.10. Assessment of Gray Mold Resistance Induced by the Tested Strains of B. bassiana in Planta

The disease resistance of detached leaves was assessed using the second leaf from the top of the tomato plants colonized by *B. bassiana*. Each treatment involved ten tomato leaves, with each treatment replicated three times. Tomato leaves of similar size were selected and disinfected. Surface sterilization was performed by sequentially immersing the leaves in a 1% (*v*/*v*) sodium hypochlorite solution, a 75% (*v*/*v*) ethanol solution, and sterile water, with each immersion lasting 30 s. The sterilized leaves were then placed in sterile glass petri dishes with a diameter of 9.0 cm, lined with filter paper. Excess water on the leaf surface was absorbed using absorbent paper. A 3 mm diameter mycelial plug was obtained from the pathogen culture medium and placed mycelium-side down on one side of the midvein. Moist, sterile cotton was added at the petiole to prevent leaf desiccation. The sealed dishes were then placed in a 27 °C incubator. The disease condition of the leaves was observed every 24 h for six consecutive days. At 6 d post-inoculation (dpi), the disease incidence in the tomato plants was calculated as follows [31,34]:

$$Incidence\;rate(\%)=(\frac{Number\;of\;plants\;with\;disfigured\;spots}{Total\;number\;of\;tomato\;plants})\times 100\%$$

The diameter of the resulting lesions was assessed after the disease spots appeared on the tomato leaves, using a ruler. Measurements were taken from the center of the inoculation block, recording both the horizontal and vertical diameters. The average of the two measurements was calculated to determine the overall diameter of the lesions. Additionally, photographs were taken to document the morphology of the lesions.

### 2.11. Statistical Analysis

Data were tested for normality assumptions using qqplot before analysis. Levene’s homogeneity test and Shapiro–Wilk normality test were set at the 0.05 significance level. We used one-way ANOVA to test the difference in conidia yield, germination rate of conidia, and average diameter of the colony of *B. bassiana* between the four treatments; *B. bassiana* virulence was estimated by the Kaplan Meier method. Multiple comparisons of mean values were made using a Tukey’s test (*p* < 0.05). We used a one-way ANOVA to test the difference in effects between two types of *B. bassiana* infective units in tomato by the diameter of ensuing lesion(s) and *B. cinerea* incidence rates. Significant differences were determined with Duncan’s multiple range test. All statistical analyses were performed using SPSS 17.0 (SPSS Inc., 2008) [23,35].

## 3. Results

### 3.1. Construction of the Vector

**Figure 1:**
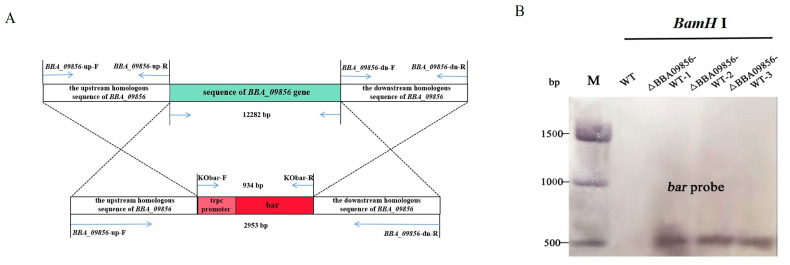
Validation of the recombinant vector. (**A**) Gene knockout schematic diagram. (**B**) Southern blot hybridization of the three ∆*BBA09856*-WT mutants and the wild type using a bar probe. Genomic DNA was digested with *BamH*I. Sizes of DNA markers (Trans 15 K) are shown in bp on the left.

PCR amplifications were carried out using the primers *BBA09856*-up-F and *BBA09856*-dn-R, with positive plasmids from *E.coli* or *A. tumefaciens* as the templates. The resulting product measured 2953 bp, which corresponded to the expected fragment size (Figure 1A). Subsequent sequencing confirmed the accuracy of the cloned fragment, which was designated pDHt-*BBA09856*-up-bar-*BBA09856*-dn.

### 3.2. Screening of Transformants

Using the primers Kobar-F and Kobar-R, a 934 bp fragment containing the trpc promoter sequence and the bar sequence was identified in the transformed *B. bassiana* (∆*BBA09856*-WT). The size of the fragment corresponded to the expected length, and the sequencing results displayed stable peak patterns without overlapping peaks. The PCR validation sequencing chromatograms of the three mutants are shown in Figure S1. Southern blot hybridization further demonstrated that the bar probe hybridized to a single band of 550 bp in the three mutant strains ∆*BBA09856*-WT, while no hybridization to the target band was observed in the wild type (Figure 1B).

### 3.3. The Biological Characteristics of the Mutant ∆BBA09856-WT

**Figure 2:**
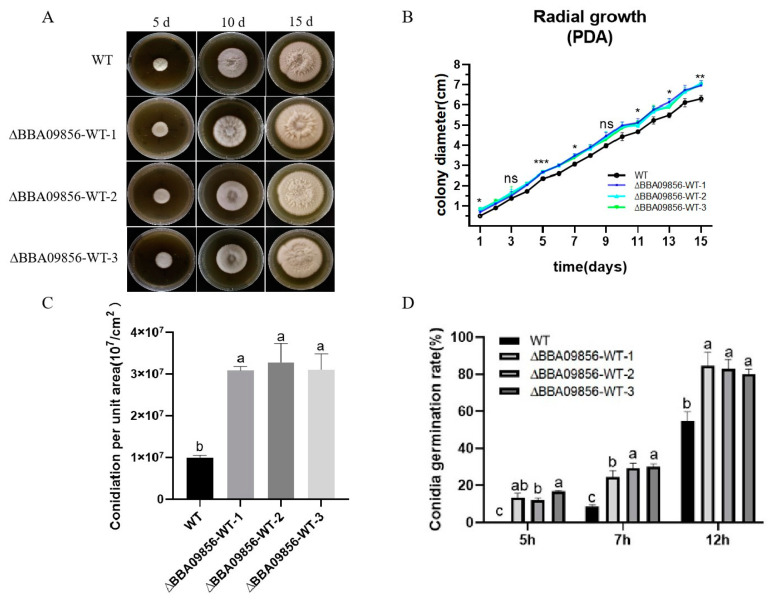
Biological characteristics of the different *B. bassiana* strains. (**A**) The phenotype of colony growth. (**B**) Colony diameter. (**C**) Conidial production of the tested strains of *B. bassiana*. (**D**) Conidia germination rates of the tested strains of *B. bassiana*. Values are means ± SE. Different letters above bars indicate significant differences between the four treatments at *p* < 0.05; ns indicates no significant difference; * indicates significant difference (*p* < 0.05); ** indicates significant difference (*p* < 0.01); and *** indicates significant difference (*p* < 0.001).

In terms of macromorphology, no significant differences were observed between the three mutant strains of ∆*BBA09856*-WT and the wild-type (WT) strain during the early developmental stages. However, in the later stages, the surfaces of all three mutant strains of ∆*BBA09856*-WT exhibited a pale yellow appearance (Figure 2A). During the 15 d of culture, there was no significant difference in the growth rate of the three mutant strains of ∆*BBA09856*-WT compared to that of the wild-type (WT) strain in the initial phase. However, the growth rate increased significantly in the later stages (Figure 2B). The spore production rates of the three mutant strains of ∆*BBA09856*-WT were significantly higher than that of the WT strain (3.09 × 10^7^/cm^2^, 3.28 × 10^7^/cm^2^, 3.11 × 10^7^/cm^2^ vs. 9.95 × 10^6^/cm^2^, *p* < 0.001) (Figure 2C). The spores of all the mutant strains of ∆*BBA09856*-WT germinated 2 h earlier than those of the WT strain, which germinated after 7 h in a shaking culture. After 12 h of shaking culture, the three mutant strains of ∆*BBA09856*-WT exhibited spore germination rates of 84.53%, 82.78%, and 80.20%, respectively, in contrast to the WT strain, which displayed a germination rate of 54.72% (Figure 2D). These results indicated that the three mutant strains ∆*BBA09856*-WT had a significantly higher germination rate (*p* < 0.001).

### 3.4. Virulence of the Mutant Strain ∆BBA09856-WT Toward O. furnacalis

**Figure 3:**
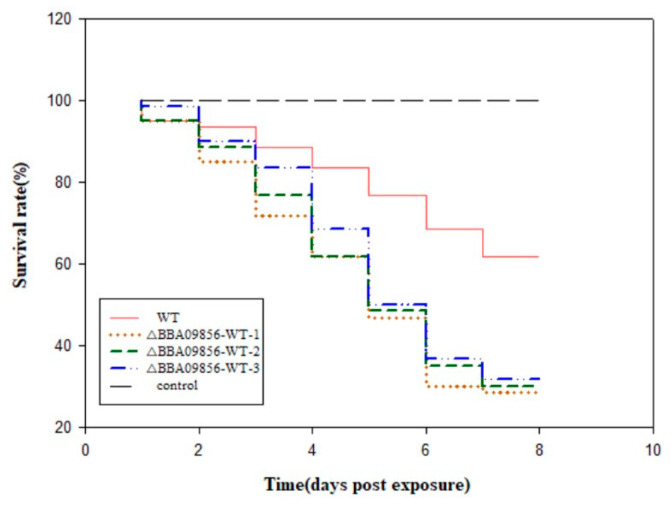
Virulence of the tested *B. bassiana* strains on *O. furnacalis* in the laboratory.

Utilizing conidia to infect third-instar larvae of *O. furnacalis* demonstrated a declining trend in the accumulated survival rates of these larvae over time (Figure 3). The survival rates of *O. furnacalis* infected by the WT strain and the three mutant strains of ∆*BBA09856*-WT were 61.66% vs. 28.33%, 30.00%, and 31.67%, respectively, after 8 d. The pathogenicity of the three mutant strains of ∆*BBA09856*-WT was significantly higher than that of the WT strain. This finding indicated that the pathogenicity of the three mutant strains of ∆*BBA09856*-WT towards the third-instar larvae of *O. furnacalis* was significantly higher than that of the WT strain. Furthermore, *O. furnacalis* exhibited muscardine cadavers on the fifth day of infection with the three mutant strains of ∆*BBA09856*-WT, whereas muscardine cadavers appeared on the seventh day with the wild-type strain WT.

### 3.5. The Antagonistic Activity of the ∆BBA09856-WT Mutant Against B. cinerea

**Figure 4:**
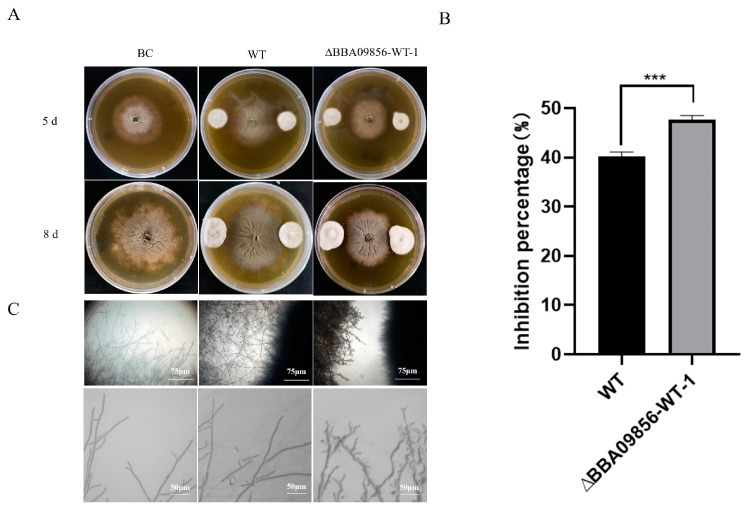
Antagonistic activity of the tested strains of *B. bassiana* against *B. cinerea*. (**A**) The plate confrontation tests of the inhibitory effects of the assays strains on the growth of *B. cinerea.* (**B**) The inhibition percentage of the tested strains of *B. bassiana* on *B. cinerea*. (**C**) The effect of the tested strains of *B. bassiana* on the hyphal morphology of *B. cinerea*. *** indicates significant difference (*p* < 0.001). BC, *B. cinerea* control.

The results of the dual culture test indicated that the mutant strain ∆*BBA09856*-WT exhibited a stronger antagonistic effect against the gray mold pathogen, *B. cinerea*, compared with that of the WT strain (Figure 4A). The inhibitory rates of the WT strain and the mutant strain ∆*BBA09856*-WT against *B. cinerea* were 40.25% and 47.65%, respectively (*p* < 0.001) (Figure 4B). Microscopic observations revealed that the mutant strain ∆*BBA09856*-WT significantly altered the apical morphology of *B. cinerea* hyphae, resulting in a visibly shriveled and thickened appearance (Figure 4C).

### 3.6. Secondary Metabolites from B. bassiana Inhibited the Growth of B. cinerea

**Figure 5:**
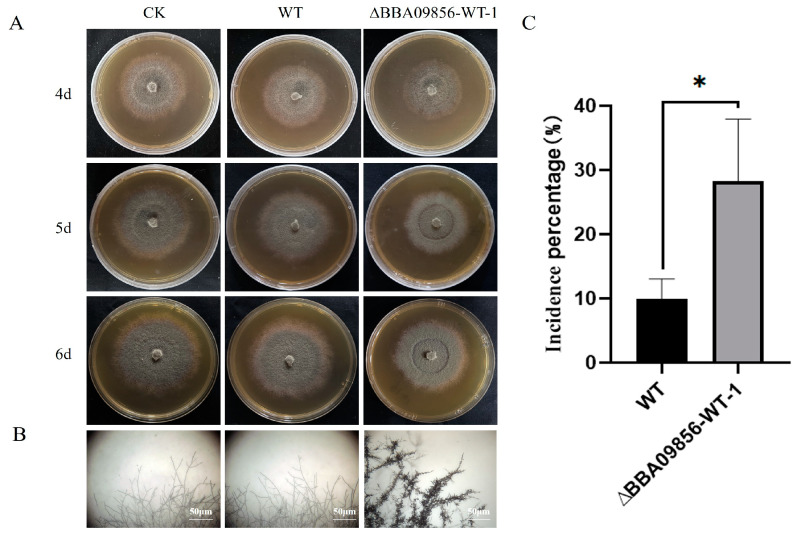
The inhibitory effect of the secondary metabolites of the tested strains of *B. bassiana* on *B. cinerea*. (**A**) The inhibitory effects of the secondary metabolites of the tested strains of *B. bassiana* on the growth of *B. cinerea*. (**B**) The effect of the secondary metabolites of the tested strains of *B. bassiana* on the hyphal morphology of *B. cinerea*. (**C**) The inhibition percentage of secondary metabolites of the tested strains of *B. bassiana* on *B. cinerea*. * indicates significant difference (*p* < 0.05).

From the plate test, both the secondary metabolites of the WT strain and the mutant strain ∆*BBA09856*-WT demonstrated an inhibitory effect on the growth of *B. cinerea* (Figure 5A). Similar to the dual culture test, the metabolites of the mutant strain ∆*BBA09856*-WT also influenced the apical morphology of the hyphae of *B. cinerea*, resulting in noticeable shrinkage and thickening of the hyphae (Figure 5B). The WT strain reduced *B. cinerea* growth by 9.90%, while the mutant strain ∆*BBA09856*-WT exhibited a significantly greater inhibition rate of 28.29% (*p* < 0.05) (Figure 5C). This suggested that deletion of the *BBA_09856* gene enhanced the antagonistic effect of the mutant strain against *B. cinerea* by increasing the production of secondary metabolites in *B. bassiana.*

### 3.7. Impact on the In Vitro Leaf Defense Effectiveness Against B. cinerea

**Figure 6:**
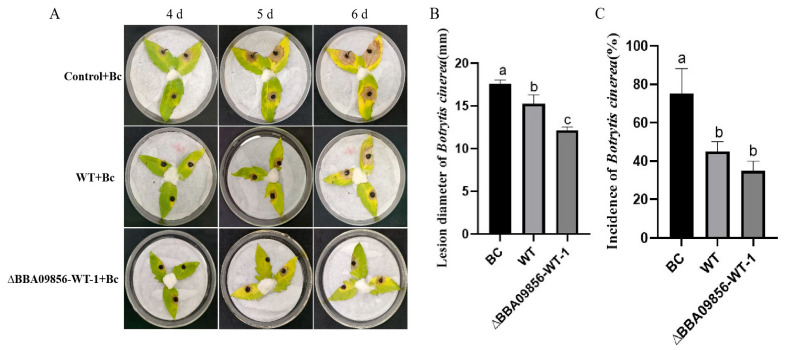
Control efficacy of the tested strains of *B. bassiana* on *B. cinerea* in vitro in tomato leaves. (**A**) The control efficacy of the tested strains of *B. bassiana* on *B. cinerea* in vitro in tomato leaves. (**B**) Effects of the tested strains of *B. bassiana* on the diameter of the gray mold lesions. (**C**) Effects of the tested strains of *B. bassiana* on the incidence of gray mold. Values are means ± SE. Different letters above bars indicate significant differences between the three treatments at *p* < 0.05.

*Botrytis cinerea* was inoculated onto tomato leaves subjected to different treatments. By the fourth day post-inoculation with *B. cinerea*, all treatments exhibited distinct levels of disease symptoms. Among them, the tomato leaves treated with the germinating spores of the mutant strain ∆*BBA09856*-WT displayed relatively fewer disease symptoms and a lower disease incidence compared with those of the WT strain. As the area of disease spots continued to expand, by the fifth day, the disease spot areas on the tomato leaves in the control group, which was solely inoculated with gray mold, had reached approximately 40–50% of the entire leaf surface. On the sixth day, it was observed that the leaves began to decay, and the affected area gradually expanded to cover the entire leaf. Meanwhile, the leaves treated with the WT strain and the mutant strain ∆*BBA09856*-WT exhibited disease spots that covered only about 40–50% of the leaf (Figure 6A).

The average diameters of *B. cinerea* disease lesions in the treatment groups Bc (*B. cinerea*), WT+Bc, and ∆BBA09856-WT+Bc were 17.60 mm, 15.26 mm, and 12.16 mm, respectively, six days after inoculation (Figure 6B). Statistical analysis showed significant differences in lesion sizes between the WT+Bc and ∆BBA09856-WT+Bc groups (*p* < 0.05).

After the endophytic colonization of tomato plants by the germinating spores of the mutant strain ∆*BBA09856*-WT, there was an increase in the induced resistance of the tomato plants, showing delay and control of the expansion of disease spots. On the sixth day after inoculation with *B. cinerea*, the incidence of disease spots in the BC treatment group was 75.00%, the incidence in the WT treatment group was 45.00%, and the incidence in the ∆*BBA09856*-WT group was 35.00%. Both *B. bassiana* treatment groups significantly reduced the incidence of gray mold. However, there was no significant difference between the WT and ∆*BBA09856*-WT treatment groups (Figure 6C).

## 4. Discussion

Fungal PKS–NRPS hybrid enzymes, whose structure consists of an iterative type I PKS followed by a single module NRPS, produce a wide variety of structurally diverse secondary metabolites including polyketide–amino acid hybrids [36,37,38,39,40,41]. In the typical fungal PKS–NRPS, the PKS portion consists of KS, AT, and ACP domains, along with several modifying domains such as KR, DH, and methyltransferase domains. The NRPS portion consists of adenylation (A), thiolation (T), also known as PCP for peptidyl carrier protein), condensation (C), and terminal release or cyclization (R), reductase or DKC, Dieckmann cyclization) domains [36,42]. Analysis by Liu et al. demonstrates that the genome of *B. bassiana* ARSEF 2860 contains twenty-one NRPS or NRPS-like genes and three PKS–NRPS hybrid genes [18,43]. Among these, the *BBA_04327* gene resembles the TAS1 NRPS–PKS hybrid enzyme synthesizing TeA [36]. Yun et al. constructed the OSM1 (MGG_01822) knockout strains of *Magnaporthe oryzae* (Dosm1) and analyzed the metabolites produced by the mutant strain Dosm1. As a result, the strain Dosm1 produced the metabolite TeA, which is synthesized by the TAS1 NRPS–PKS [36]. Previous research indicates that Bacillaene is synthesized by PKS–NRPS, a hybrid PK/NRP natural product from Bacillus subtilis [44,45,46]. The PKS–NRPS gene, *BBA09856*, investigated in this study has a length of 12,282 bp and encodes a PKS–NRPS hybrid protein that contains the domains of KS, AT, DH, C-Met, C (Condensation), A (Adenylation), and T (Thiolation) [18]. The AT and KS domains are responsible for selecting acyl-CoA building blocks and extending polyketide intermediates. Additionally, the DH domain is involved in the further processing of the β-ketoacyl-ACP product resulting from KS extension. Moreover, the AT or C-Met domains can introduce methyl groups at the α-position of a polyketide intermediate through the incorporation of methylmalonyl-CoA extender units. Although recent studies have elucidated the significance of *pks* and *nrps* genes, emphasizing their diverse roles in fungal development [19,47], environmental adaptation [3], and potential for biocontrol against insect pests in *B. bassiana* [22], the biological functions of their hybrid protein have remained unclear.

Herein, we found that the mutant strain with the knockout of the PKS–NRPS gene *BBA09856* showed no significant change in early growth rate compared with that of the WT strain, but exhibited a significantly increased growth rate in later stages. The spores from the mutant strain ∆*BBA09856*-WT germinated earlier than those from the WT strain, with a notable difference in germination rates evident at the 12 h mark. The mutant strain ∆*BBA09856*-WT demonstrated a markedly higher conidiation rate per unit area, along with a significant enhancement in its virulence against *O. furnacalis* larvae. These results suggest that the PKS–NRPS gene *BBA_09856* correlates negatively with the growth and development of *B. bassiana*, as well as with the germination and sporulation of conidia and its virulence toward *O. furnacalis*. Yang et al. demonstrated that PKS and NRPS exhibit inherent substrate selectivity and engage in competitive substrate binding among their different catalytic domains [48]. The insertion, deletion, or substitution of these catalytic domains might lead to the production of novel polyketide derivatives or the accumulation of desired products [49]. It is possible that the binding domains, such as KS, AT, DH and Met in the PKS–NRPS protein encoded by the *BBA_09856* gene in this study compete with other functional PKS or NRPS for substrate binding. And, the efficiency of the PKS–NRPS catalytic site may be relatively low due to the influence of the hybrid protein. When the *BBA_09856* gene was knocked out, there was no PKS–NRPS available to compete with other highly efficient PKS or NRPS for substrates. This resulted in the promotion of *B. bassiana* growth, enhanced conidial germination, and increased toxin synthesis, ultimately leading to greater virulence of the mutant strain against *O. furnacalis*.

It has been demonstrated that the insect pathogens *Beauveria* spp. and *Metarhizium* spp. are evolutionarily diverged from plant endophytes or pathogens [19,47,50]. According to reports, *Beauveria* species have insecticidal properties and can also act as an endophytic fungi in plants [19]. Sui et al. reported that colonization by *B. bassiana* significantly mitigated the incidence of plant diseases caused by *Botrytis cinerea* [31]. Ownley et al. first reported that colonization by the *B. bassiana* strain 11–98 could effectively suppress damping-off disease in tomatoes, which is caused by the soil-borne pathogens *Rhizoctonia solani* and *Pythium myriotylum* [51]. Furthermore, pre-treatment of cotton seedlings with the same *B. bassiana* strain resulted in a reduction in the severity of bacterial blight caused by *Xanthomonas axonopodis* pv. malvacearum (Xam) [52]. There is a growing body of evidence suggesting that certain endophytic fungal entomopathogens possess antagonistic properties against plant pathogens, thereby mitigating their detrimental effects on host plants [53,54]. As reported by Nisa et al., many endophyte species produce a variety of metabolites within plants [55]. Gurulingappa et al. hypothesized that endophytic colonization by *Lecanicillium lecanii* and *B. bassiana* could lead to the accumulation of fungal toxins and metabolites within plant tissues, thereby affecting the survival and reproduction of *Aphis gossypii* [56]. Colonization of cotton leaves by *Lecanicillium lecanii* or *B. bassiana* resulted in a notable decrease in the reproduction of *Aphis gossypii*, while colonization of wheat leaves by *B. bassiana* led to a significant reduction in the weight of *Chortoicetes terminifera* [57]. The mortality of adult *Cosmopolites sordidus* and the subsequent reduction in larval damage were attributed to the successful establishment of *B. bassiana* within banana plants [58]. The findings of the present study indicated that knockout of the *BBA09856* gene significantly enhanced both the hyphal growth and conidial germination rate of *B. bassiana*. This enhancement could potentially boost the ability of *B. bassiana* to stimulate disease resistance in tomato.

Feng et al. identified the antimicrobial compound oosporein from *B. bassiana*, which is a product of diphenyl quinone [19,59,60,61,62]. Oosporein is synthesized through the polyketide synthase pathway in *B. bassiana* [19]. Additionally, various plant pathogenic fungi and endophytic fungi can produce oosporein, which exhibits antimicrobial activities against fungi, bacteria, and other microorganisms [60,63,64]. Additional research indicates that numerous endophytic fungi are capable of producing various metabolites [65]. *Bacillus subtilis* FA26 produces secondary metabolites that disrupt the hyphal structure of *Clavibacter michiganensis* and alter the morphology of colonies, hyphae, and spores [66]. Our research found that the metabolites produced by mutant strain ∆*BBA09856*-WT could significantly alter the mycelial morphology of *B. cinerea*. Additionally, the mutant strain exhibited markedly higher inhibitory activity against *B. cinerea* compared with that of the WT strain. In vitro test results indicated that tomatoes colonized by the mutant strain demonstrated significantly greater resistance to gray mold than those colonized by the WT strain. Moreover, research indicates that specific domains of PKS, such as C-methyltransferase (C-MeT), may have the capacity to either accept or reject different substrates provided by the acyl carrier protein (ACP), suggesting the possibility of competition among these domains for substrates [67]. Based on existing research, it can be inferred that the knockout of the *BB_A09856* gene in *B. bassiana* could lead to a deficiency of the heterologous protein PKS–NRPS, which competes with other PKSs for substrates. Consequently, this deficiency promotes the synthesis of secondary metabolites by other PKSs, thereby enhancing the resistance of plants endophytically colonized by the *B. bassiana* to plant diseases. However, the influence of the *BBA09856* gene on the different types of metabolites produced by *B. bassiana*, as well as its effects on other characteristics of the fungus, including resistance to desiccation, UV light, and spore survival, necessitates further investigation.

## 5. Conclusions

This study successfully engineered a mutant of *B. bassiana* with a deletion of the PKS–NRPS gene *BBA_09856*, yielding a genetically stable mutant strain, Δ*BBA09856*-WT. Deletion of the gene *BBA_09856* in *B. bassiana* significantly improved the growth rate, germination rate, and spore production compared with those of the WT strain. Additionally, this genetic alteration enhanced the virulence of *B. bassiana* against *O. furnacalis* larvae and increased its antagonistic effects against the plant pathogenic fungus *B. cinerea*. The in vitro experimental results indicated that tomato plants colonized by the mutant ∆*BBA09856*-WT exhibited significantly higher resistance to *B. cinerea* compared with those colonized by the WT strain. Therefore, this study found that the *BBA_09856* gene plays multiple physiological roles in the growth and virulence of *B. bassiana.*

## Data Availability

The original contributions presented in this study are included in the article and Supplementary Material. Further inquiries can be directed to the corresponding authors.

## Supplementary Materials

The following supporting information can be downloaded at: https://www.mdpi.com/article/10.3390/jof11030197/s1, Table S1: Sequences of primers used in the present study; Figure S1: Using the primers KObar-F and KObar-R, a 934 bp fragment containing the trpc promoter sequence and the bar sequence was identified in the transformed *B. bassiana* (∆*BBA09856*-WT).

## References

[1] Altimira F., Arias-Aravena M., Jian L., Real N., Correa P., González C., Godoy S., Castro J.F., Zamora O., Vergara C. (2022). Genomic and Experimental Analysis of the Insecticidal Factors Secreted by the Entomopathogenic Fungus *Beauveria pseudobassiana* RGM 2184. J. Fungi.

[2] Paschapur A., Subbanna A.R.N.S., Singh A.K., Jeevan B., Mishra K.K. (2021). Unraveling the Importance of Metabolites from Entomopathogenic Fungi in Insect Pest Management. Microbes for Sustainable lnsect Pest Management. Sustainability in Plant and Crop Protection.

[3] Meng X., Liao Z., Liu T., Hussain K., Chen J., Fang Q., Wang J. (2021). Vital Roles of Pks11, a Highly Reducing Polyketide Synthase, in Fungal Conidiation, Antioxidant Activity, Conidial Cell Wall Integrity, and UV Tolerance of *Beauveria bassiana*. J. Invertebr. Pathol..

[4] Branine M., Bazzicalupo A., Branco S. (2019). Biology and Applications of Endophytic Insect-Pathogenic Fungi. PLoS Pathog..

[5] Mannino M.C., Huarte-Bonnet C., Davyt-Colo B., Pedrini N. (2019). Is the Insect Cuticle the Only Entry Gate for Fungal Infection? Insights into Alternative Modes of Action of Entomopathogenic Fungi. J. Fungi.

[6] Mantzoukas S., Eliopoulos P.A. (2020). Endophytic Entomopathogenic Fungi: A Valuable Biological Control Tool against Plant Pests. Appl. Sci..

[7] Wang J., Ma Y., Liu Y., Tong S., Zhu S., Jin D., Pei Y., Fan Y. (2020). A Polyketide Synthase, BbpksP, Contributes to Conidial Cell Wall Structure and UV Tolerance in *Beauveria bassiana*. J. Invertebr. Pathol..

[8] Ortiz-Urquiza A., Keyhani N.O. (2016). Molecular Genetics of *Beauveria bassiana* Infection of Insects. Adv. Genet..

[9] Fernandes É.K.K., Rangel D.E.N., Braga G.U.L., Roberts D.W. (2015). Tolerance of Entomopathogenic Fungi to Ultraviolet Radiation: A Review on Screening of Strains and Their Formulation. Curr. Genet..

[10] Wang H., Peng H., Li W., Cheng P., Gong M. (2021). The Toxins of *Beauveria bassiana* and the Strategies to Improve Their Virulence to Insects. Front. Microbiol..

[11] Wang J., Liu J., Hu Y., Ying S.-H., Feng M.-G. (2013). Cytokinesis-Required Cdc14 Is a Signaling Hub of Asexual Development and Multi-Stress Tolerance in *Beauveria bassiana*. Sci. Rep..

[12] Butt T.M., Coates C.J., Dubovskiy I.M., Ratcliffe N.A. (2016). Entomopathogenic Fungi: New Insights into Host-Pathogen Interactions. Adv. Genet..

[13] Sain S.K., Monga D., Hiremani N.S., Nagrale D.T., Kranthi S., Kumar R., Kranthi K.R., Tuteja O.P., Waghmare V.N. (2021). Evaluation of Bioefficacy Potential of Entomopathogenic Fungi against the Whitefly (*Bemisia tabaci* Genn.) on Cotton under Polyhouse and Field Conditions. J. Invertebr. Pathol..

[14] Wilcken C.F., Dal Pogetto M.H.F.d.A., Lima A.C.V., Soliman E.P., Fernandes B.V., da Silva I.M., Zanuncio A.J.V., Barbosa L.R., Zanuncio J.C. (2019). Chemical vs Entomopathogenic Control of *Thaumastocoris peregrinus* (Hemiptera: Thaumastocoridae) via Aerial Application in Eucalyptus Plantations. Sci. Rep..

[15] Altimira F., De La Barra N., Godoy P., Roa J., Godoy S., Vitta N., Tapia E. (2021). Lobesia Botrana: A Biological Control Approach with a Biopesticide Based on Entomopathogenic Fungi in the Winter Season in Chile. Insects.

[16] Zhang L., Yue Q., Wang C., Xu Y., Molnár I. (2020). Secondary Metabolites from Hypocrealean Entomopathogenic Fungi: Genomics as a Tool to Elucidate the Encoded Parvome. Nat. Prod. Rep..

[17] Marahiel M.A., Essen L.-O. (2009). Chapter 13. Nonribosomal Peptide Synthetases Mechanistic and Structural Aspects of Essential Domains. Methods Enzymol..

[18] Liu H., Xie L., Wang J., Guo Q., Yang S., Liang P., Wang C., Lin M., Xu Y., Zhang L. (2017). The Stress-Responsive and Host-Oriented Role of Nonribosomal Peptide Synthetases in an Entomopathogenic Fungus, *Beauveria bassiana*. J. Microbiol. Biotechnol..

[19] Feng P., Shang Y., Cen K., Wang C. (2015). Fungal Biosynthesis of the Bibenzoquinone Oosporein to Evade Insect Immunity. Proc. Natl. Acad. Sci. USA.

[20] Srisuksam C., Punya J., Wattanachaisaereekul S., Toopaang W., Cheevadhanarak S., Tanticharoen M., Amnuaykanjanasin A. (2018). The Reducing Clade IIb Polyketide Synthase PKS14 Acts as a Virulence Determinant of the Entomopathogenic Fungus *Beauveria bassiana*. FEMS Microbiol. Lett..

[21] Toopaang W., Phonghanpot S., Punya J., Panyasiri C., Klamchao K., Wasuwan R., Srisuksam C., Sangsrakru D., Sonthirod C., Tangphatsornruang S. (2017). Targeted Disruption of the Polyketide Synthase Gene *pks15* Affects Virulence against Insects and Phagocytic Survival in the Fungus *Beauveria bassiana*. Fungal Biol..

[22] Udompaisarn S., Toopaang W., Sae-Ueng U., Srisuksam C., Wichienchote N., Wasuwan R., Nahar N.A.S., Tanticharoen M., Amnuaykanjanasin A. (2020). The Polyketide Synthase PKS15 Has a Crucial Role in Cell Wall Formation in *Beauveria bassiana*. Sci. Rep..

[23] Sui L., Lu Y., Zhu H., Wan T., Li Q., Zhang Z. (2022). Endophytic Blastospores of *Beauveria bassiana* Provide High Resistance against Plant Disease Caused by *Botrytis cinerea*. Fungal Biol..

[24] Tefera T., Vidal S. (2009). Effect of Inoculation Method and Plant Growth Medium on Endophytic Colonization of Sorghum by the Entomopathogenic Fungus *Beauveria bassiana*. Biocontrol.

[25] Parsa S., Ortiz V., Vega F.E. (2013). Establishing Fungal Entomopathogens as Endophytes: Towards Endophytic Biological Control. J. Vis. Exp..

[26] Bing L.A., Lewis L.C. (1991). Suppression of *Ostrinia nubilalis* (Hübner) (Lepidoptera: Pyralidae) by Endophytic *Beauveria bassiana* (Balsamo) Vuillemin. Environ. Entomol..

[27] Cherry A.J., Banito A., Djegui D., Lomer C. (2004). Suppression of the Stem-Borer *Sesamia calamistis* (Lepidoptera; Noctuidae) in Maize Following Seed Dressing, Topical Application and Stem Injection with African Isolates of *Beauveria bassiana*. Pans Pest. Artic. News Summ..

[28] Pinnamaneni R., Kalidas P., Sambasiva Rao K.R.S. (2011). Studies on the Cloning and Expression of *Bbchit1* Gene of *Beauveria bassiana* NCIM 1216. Indian J. Microbiol..

[29] Cen K., Li B., Lu Y., Zhang S., Wang C. (2017). Divergent LysM Effectors Contribute to the Virulence of *Beauveria bassiana* by Evasion of Insect Immune Defenses. PLoS Pathog..

[30] Fang W., Zhang Y., Yang X., Zheng X., Duan H., Li Y., Pei Y. (2004). Agrobacterium Tumefaciens-Mediated Transformation of *Beauveria bassiana* Using an Herbicide Resistance Gene as a Selection Marker. J. Invertebr. Pathol..

[31] Sui L., Lu Y., Zhou L., Li N., Li Q., Zhang Z. (2023). Endophytic *Beauveria bassiana* Promotes Plant Biomass Growth and Suppresses Pathogen Damage by Directional Recruitment. Front. Microbiol..

[32] Kang Q., Ning S., Sui L., Lu Y., Zhao Y., Shi W., Li Q., Zhang Z. (2023). Transcriptomic Analysis of Entomopathogenic Fungus *Beauveria bassiana* Infected by a Hypervirulent Polymycovirus BbPmV-4. Fungal Biol..

[33] Sui L., Lu Y., Xu M., Liu J., Zhao Y., Li Q., Zhang Z. (2024). Insect Hypovirulence-Associated Mycovirus Confers Entomopathogenic Fungi with Enhanced Resistance against Phytopathogens. Virulence.

[34] Li T., Zhou J., Li J. (2023). Combined effects of temperature and humidity on the interaction between tomato and *Botrytis cinerea* revealed by integration of histological characteristics and transcriptome sequencing. Hortic. Res..

[35] Li S., Hui Z., Wenjing X., Qinfeng G., Ling W., Zhengkun Z., Qiyun L., Deli W. (2020). Elevated Air Temperature Shifts the Interactions between Plants and Endophytic Fungal Entomopathogens in an Agroecosystem. Fungal Ecol..

[36] Yun C.-S., Motoyama T., Osada H. (2015). Biosynthesis of the Mycotoxin Tenuazonic Acid by a Fungal NRPS-PKS Hybrid Enzyme. Nat. Commun..

[37] Fisch K.M. (2013). Biosynthesis of Natural Products by Microbial Iterative Hybrid PKS–NRPS. RSC Adv..

[38] Boettger D., Hertweck C. (2013). Molecular Diversity Sculpted by Fungal PKS-NRPS Hybrids. ChemBioChem.

[39] Böhnert H.U., Fudal I., Dioh W., Tharreau D., Notteghem J.-L., Lebrun M.-H. (2004). A Putative Polyketide Synthase/Peptide Synthetase from *Magnaporthe grisea* Signals Pathogen Attack to Resistant Rice. Plant Cell.

[40] Song Z., Cox R.J., Lazarus C.M., Simpson TJ T.J. (2004). Fusarin C Biosynthesis in *Fusarium moniliforme* and *Fusarium venenatum*. ChemBioChem.

[41] Eley K.L., Halo L.M., Song Z., Powles H., Cox R.J., Bailey A.M., Lazarus C.M., Simpson T.J. (2007). Biosynthesis of the 2-Pyridone Tenellin in the Insect Pathogenic Fungus *Beauveria bassiana*. ChemBioChem.

[42] Hertweck C., Luzhetskyy A., Rebets Y., Bechthold A. (2007). Type II Polyketide Synthases: Gaining a Deeper Insight into Enzymatic Teamwork. Nat. Prod. Rep..

[43] Pedrini N. (2022). The Entomopathogenic Fungus *Beauveria bassiana* Shows Its Toxic Side within Insects: Expression of Genes Encoding Secondary Metabolites during Pathogenesis. J. Fungi.

[44] Patel P.S., Huang S., Fisher S., Pirnik D., Aklonis C., Dean L., Meyers E., Fernandes P., Mayerl F. (1995). Bacillaene, a Novel Inhibitor of Procaryotic Protein Synthesis Produced by *Bacillus subtilis*: Production, Taxonomy, Isolation, Physico-Chemical Characterization and Biological Activity. J. Antibiot..

[45] Albertini A.M., Caramori T., Scoffone F., Scotti C., Galizzi A. (1995). Sequence around the 159 Degree Region of the *Bacillus subtilis* Genome: The pksX Locus Spans 33.6 Kb. Microbiology.

[46] Butcher R.A., Schroeder F.C., Fischbach M.A., Straight P.D., Kolter R., Walsh C.T., Clardy J. (2007). The Identification of Bacillaene, the Product of the PksX Megacomplex in *Bacillus subtilis*. Proc. Natl. Acad. Sci. USA.

[47] Xiao G., Ying S.H., Zheng P., Wang Z.L., Zhang S., Xie X.Q., Shang Y., St. Leger R.J., Zhao G.-P., Wang C. (2012). Genomic Perspectives on the Evolution of Fungal Entomopathogenicity in *Beauveria bassiana*. Sci. Rep..

[48] Yang X.-L., Friedrich S., Yin S., Piech O., Williams K., Simpson T.J., Cox R.J. (2019). Molecular Basis of Methylation and Chain-Length Programming in a Fungal Iterative Highly Reducing Polyketide Synthase. Chem. Sci..

[49] Wang H., Liang J., Yue Q., Li L., Shi Y., Chen G., Li Y.-Z., Bian X., Zhang Y., Zhao G. (2021). Engineering the Acyltransferase Domain of Epothilone Polyketide Synthase to Alter the Substrate Specificity. Microb. Cell Fact..

[50] Hu X., Xiao G., Zheng P., Shang Y., Su Y., Zhang X., Liu X., Zhan S., St Leger R.J., Wang C. (2014). Trajectory and Genomic Determinants of Fungal-Pathogen Speciation and Host Adaptation. Proc. Natl. Acad. Sci. USA.

[51] Ownley B.H., Pereira R.M., Klingeman W.E., Quigley N.B., Leckie B.M. (2004). Beauveria bassiana, a Dual Purpose Biocontrol Organism, with Activity against Insect Pests and Plant Pathogens. Emerging Concepts in Plant Health.

[52] Ownley B.H., Griffin M.R., Klingeman W.E., Gwinn K.D., Moulton J.K., Pereira R.M. (2008). *Beauveria bassiana*: Endophytic Colonization and Plant Disease Control. J. Invertebr. Pathol..

[53] Barra-Bucarei L., France Iglesias A., Gerding González M., Silva Aguayo G., Carrasco-Fernández J., Castro J.F., Ortiz Campos J. (2019). Antifungal Activity of *Beauveria bassiana* Endophyte against *Botrytis cinerea* in Two Solanaceae Crops. Microorganisms.

[54] Canassa F., Esteca F.C.N., Moral R.A., Meyling N.V., Klingen I., Delalibera I. (2020). Nicolai Root Inoculation of Strawberry with the Entomopathogenic Fungi *Metarhizium robertsii* and *Beauveria bassiana* Reduces Incidence of the Twospotted Spider Mite and Selected Insect Pests and Plant Diseases in the Field. J. Pest Sci..

[55] Nisa H., Kamili A.N., Nawchoo I.A., Shafi S., Shameem N., Bandh S.A. (2015). Fungal Endophytes as Prolific Source of Phytochemicals and Other Bioactive Natural Products: A Review. Microb. Pathog..

[56] Gurulingappa P., Mcgee P.A., Sword G. (2011). Endophytic *Lecanicillium lecanii* and *Beauveria bassiana* Reduce the Survival and Fecundity of *Aphis gossypii* Following Contact with Conidia and Secondary Metabolites. Crop Prot..

[57] Gurulingappa P., Sword G.A., Murdoch G., Mcgee P.A. (2010). Colonization of Crop Plants by Fungal Entomopathogens and Their Effects on Two Insect Pests When in Planta. Biol. Control.

[58] Akello J., Dubois T., Coyne D., Kyamanywa S. (2010). Effect of Endophytic *Beauveria bassiana* on Populations of the Banana Weevil, *Cosmopolites sordidus*, and Their Damage in Tissue-Cultured Banana Plants. Entomol. Exp. Appl..

[59] Cole R.J., Kirksey J.W., Cutler H.G., Davis E.E. (1974). Toxic Effects of Oosporein from *Chaetomium trilaterale*. J. Agric. Food Chem..

[60] Wainwright M., Betts R.P., Teale D.M. (1986). Antibiotic Activity of Oosporein from *Verticillium psalliotae*. Trans. Br. Mycol. Soc..

[61] Nagaoka T., Nakata K., Kouno K., Ando T. (2004). Antifungal Activity of Oosporein from an Antagonistic Fungus against Phytophthora Infestans. Z. Naturforsch. C.

[62] Alurappa R., Bojegowda M.R.M., Kumar V., Mallesh N.K., Chowdappa S. (2014). Characterisation and Bioactivity of Oosporein Produced by Endophytic Fungus *Cochliobolus kusanoi* Isolated from *Nerium oleander* L.. Nat. Prod. Res..

[63] Brewer D., Jen W.C., Jones G.A., Taylor A. (1984). The Antibacterial Activity of Some Naturally Occurring 2,5-Dihydroxy-1,4-Benzoquinones. Can. J. Microbiol..

[64] Terry B.J., Liu W.C., Cianci C.W., Proszynski E., Fernandes P., Bush K., Meyers E. (1992). Inhibition of Herpes Simplex Virus Type 1 DNA Polymerase by the Natural Product Oosporein. J. Antibiot..

[65] Gange A.C., Koricheva J., Currie A.F., Jaber L.R., Vidal S. (2019). Meta-Analysis of the Role of Entomopathogenic and Unspecialized Fungal Endophytes as Plant Bodyguards. New Phytol..

[66] Rajer F.U., Wu H., Xie Y., Xie S., Raza W., Has T., Gao X. (2017). Volatile Organic Compounds Produced by a Soil-Isolate, *Bacillus subtilis* FA26 Induce Adverse Ultra-Structural Changes to the Cells of *Clavibacter michiganensis* ssp. Sepedonicus, the Causal Agent of Bacterial Ring Rot of Potato. Microbiology.

[67] Roberts D.M., Bartel C., Scott A., Ivison D., Simpson T.J., Cox R.J. (2017). Substrate Selectivity of an Isolated Enoyl Reductase Catalytic Domain from an Iterative Highly Reducing Fungal Polyketide Synthase Reveals Key Components of Programming. Chem. Sci..

