# Supplementary material for: The PKS–NRPS Gene BBA_09856 Deletion Mutant of Beauveria bassiana Enhanced Its Virulence Against Ostrinia furnacalis Larvae and Strengthened the Host Plant’s Resistance to Botrytis cinerea as an Endotype
Source: J Fungi (Basel). 2025 Mar 4;11(3):197. doi: 10.3390/jof11030197 (PMC11942696; doi:10.3390/jof11030197)
Supplement: Supplementary file 1 [file jof-11-00197-s001.zip › jof-3447981-supplementary.pdf]

Supporting Table:

Table S1. Sequences of primers used in the present study

Primer Name.	Primer Sequence
BBA09856-up-F	CCGGAATTCCGGGAATTGATAGCTGAGCTAG
BBA09856-up-R	GCTGCAGCCGTGTCGCATATAGGTTCCAGG
BBA09856-dn-F	CGGACTAGTCCGCTTGCTTATTAACTACG
BBA09856-dn-R	CGAGCTCGTCTCTGCAGTCCCTGATATGG
KObar-F	GTAGACAGAAGATGATATTG
KObar-R	TCATCAGATCTCGGTGACGGG

Supporting Figures:

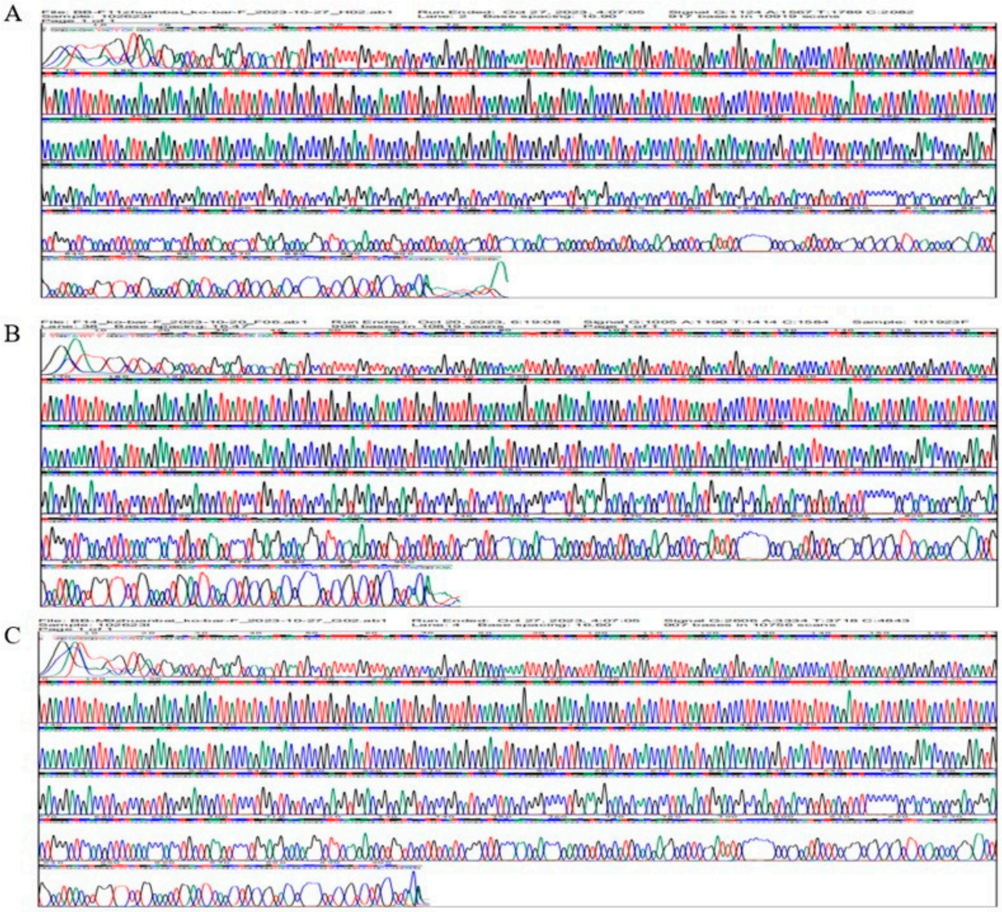

Figure S1. Using the primers KObar-F and KObar-R, a 934 bp fragment containing the trpc promoter sequence and the bar sequence was identified in the transformed *B. bassiana* (Δ*BBA09856*-WT). The size of the fragment corresponded to the expected length, and the sequencing results displayed stable peak patterns without overlapping peaks. (A): Δ*BBA09856*-WT-1; (B): Δ*BBA09856*-WT-2;(C): Δ*BBA09856*-WT-3.
